# A thermodynamic study of the cadmium–neodymium system

**DOI:** 10.1007/s00706-016-1670-5

**Published:** 2016-03-09

**Authors:** Barbara Skołyszewska-Kühberger, Thomas L. Reichmann, Herbert Ipser

**Affiliations:** Department of Inorganic Chemistry (Materials Chemistry), University of Vienna, 1090 Vienna, Austria; Karlsruhe Institute of Technology, Institute for Applied Materials, Applied Materials Physics, 76344 Eggenstein-Leopoldshafen, Germany

**Keywords:** Cd–Nd, Alloys, Lanthanoids, Thermochemistry, Vapor pressure measurements

## Abstract

**Abstract:**

Cd vapor pressures were determined over Cd–Nd samples by an isopiestic method. The measurements were carried out in the temperature range from about 690 to 1200 K and over a composition range between 48 and 92 at % Cd. From the vapor pressures, thermodynamic activities of Cd were derived for all samples at their respective sample temperatures, and partial molar enthalpies of Cd were obtained from the temperature dependence of the activities. With these partial molar enthalpies, the Cd activities were converted to a common temperature of 873 K. By means of a Gibbs–Duhem integration Nd activities and integral Gibbs energies were calculated, using a literature value of Δ_f_*G* for the phase Cd_6_Nd as integration constant. A minimum of Δ_f_*G* ≈ −38 kJ g-atom^−1^ at 873 K was obtained for the phase CdNd, a value that compares well with other CdRE compounds.

**Graphical abstract:**

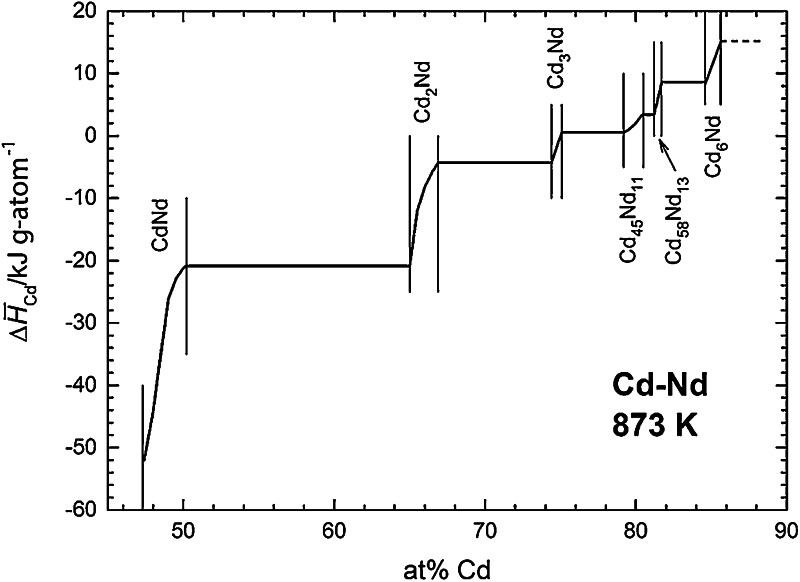

## Introduction

Although nuclear energy is phased out in some countries many others still have to rely on nuclear power to provide the necessary electrical energy. One of the key issues for future use of nuclear energy, besides reactor safety, is a reliable waste management. At present several different reprocessing techniques are known. Traditional aqueous methods suffer from some drawbacks like limited solubility of fuel materials in acidic aqueous solutions and poor radiation stability of the organic solvents employed in the extraction process [1]. The so-called pyrochemical separation techniques appear to be more efficient methods for reprocessing of spent high burn-up fuels. The central step in these non-aqueous methods is the electro-refining process where in an electro transportable cell chopped fuel rods are reprocessed [2]. This electro transportable cell contains a steel anode in form of a basket, where spent fuels are inserted, and two different cathodes: a stainless steel cathode for the recovery of U and a liquid metal cathode (using Al [1], Bi [3], or Cd [3]) for the selective recovery of Pu and minor actinides (MA). The entire cell is completely filled with a liquid LiCl–KCl electrolyte with an additional pool of liquid metal at the bottom. A variety of liquid metals like Al [1], Bi [3], or Cd [3] have been explored for the extraction of the rare earth (RE) elements (in particular, light rare earth elements between La and Gd except Pm) which are partially oxidized, out of the electrolyte. The extraction behavior is primarily affected by the formation of intermetallic compounds, which makes a thorough knowledge of the various binary RE-metal systems imperative. The existence of intermetallic compounds as well as thermodynamic properties such as their stability is of considerable interest, both for a thermodynamic assessment of the corresponding binary system based on the CALPHAD^1^ method [4] and also for an optimization of the extraction process itself.

This was the starting point for the present study which wants to provide partial thermodynamic properties of binary Cd–Nd alloys, mainly based on Cd vapor pressure measurements according to an isopiestic method [5, 6]. Using a value of the Gibbs energy of formation of the phase Cd_6_Nd that had been obtained by a CALPHAD-type optimization [7], an estimate of the integral Gibbs energies of formation could be obtained over a large composition range.

### Literature review: phase diagram

Early experimental studies of the Cd–Nd phase diagram were done by Iandelli [8, 9], Johnson et al. [10, 11], and Bruzzone et al. [12]. Based on this experimental work and an earlier assessment by Gschneidner and Calderwood [13], a rudimentary phase diagram was published in Massalski’s handbook [14]. Only very recently, the phase equilibria in the Cd–Nd system were studied in detail by Skołyszewska-Kühberger et al. [15]. Altogether seven intermetallic phases were identified: CdNd, Cd_2_Nd, and Cd_45_Nd_11_ with congruent melting behavior, and Cd_3_Nd, Cd_58_Nd_13_, Cd_6_Nd, and Cd_11_Nd with peritectic decomposition reactions. For the compound Cd_2_Nd, a transition into a high-temperature modification was found. In addition, the maximum solid solubility of Cd in α-Nd and β-Nd was determined with about 3 and 19 at %, respectively.

### Literature review: thermochemical data

Only limited thermochemical information has been available for the Cd–Nd system. Koyama et al. [16] determined the activity coefficient of Nd in liquid Cd at infinite dilution at 723 K from an investigation of the distribution of Nd between Cd(l) and a liquid chloride salt. Similarly, Kurata et al. [3, 17] employed electrochemical measurements to determine the distribution behavior of Nd between a eutectic liquid LiCl–KCl mixture and Bi(l) or Cd(l). From the results they derived the activity coefficient of Nd in Cd(l) at 773 K. Based on this limited experimental information, Kurata and Sakamura [7] performed a CALPHAD-type optimization of the Cd-rich part of the Cd–Nd system and provided calculated Gibbs energy of formation values for the phases Cd_11_Nd and Cd_6_Nd.

Recently, Vandarkuzhali et al. [18] investigated the electrochemical behavior of NdCl_3_ at a liquid Cd electrode and derived the Gibbs energy of formation of Cd_11_Nd in the temperature range between 698 and 773 K. In addition, an overview of the thermodynamic properties of actinides and RE fission products (among them also Nd) in liquid Cd was provided by Zhang et al. [19], apparently without knowledge of Ref. [18].

## Results and discussion

### Isopiestic measurements

Six successful isopiestic experiments were carried out for the Cd–Nd system, with reservoir temperatures between 687 and 893 K corresponding to total vapor pressures of Cd between about 2 and 150 mbar, respectively. The corresponding sample temperatures were between 688 and 1192 K. Since the vapor pressure of Nd is several orders of magnitude lower compared to that of Cd it can be neglected, and it can be assumed that the total pressure in the system is due to Cd maintained at a constant temperature in the reservoir. When the final equilibrium is reached in an isopiestic experiment the partial pressure of Cd over each sample at its sample temperature *T*_S_, *p*_Cd_(*T*_S_), is equal to the vapor pressure of pure Cd at the reservoir temperature $T_{\text{R}} ,p_{\text{Cd}}^{0} \left( {T_{\text{R}} } \right)$:

1 $$p_{\text{Cd}} (T_{\text{S}} ) = p_{\text{Cd}}^{0} (T_{\text{R}} )$$

Under these circumstances, the Cd activity in the samples can be calculated by the following equation:

2 $$a_{\text{Cd}} (T_{\text{S}} ) = \frac{{p_{\text{Cd}} (T_{\text{S}} )}}{{p_{\text{Cd}}^{0} (T_{\text{S}} )}} = \frac{{p_{\text{Cd}}^{0} (T_{\text{R}} )}}{{p_{\text{Cd}}^{0} (T_{\text{S}} )}}$$

The vapor pressure of pure Cd as a function of temperature was taken from Binnewies and Milke [20]:

3 $$\log \left( {\frac{{p_{\text{Cd}}^{0} }}{\text{bar}}} \right) = 8.7 - 5690 \times \frac{\text{K}}{T} - 1.07\, \times \,{ \log }\frac{T}{\text{K}}$$

**Fig. 1:**
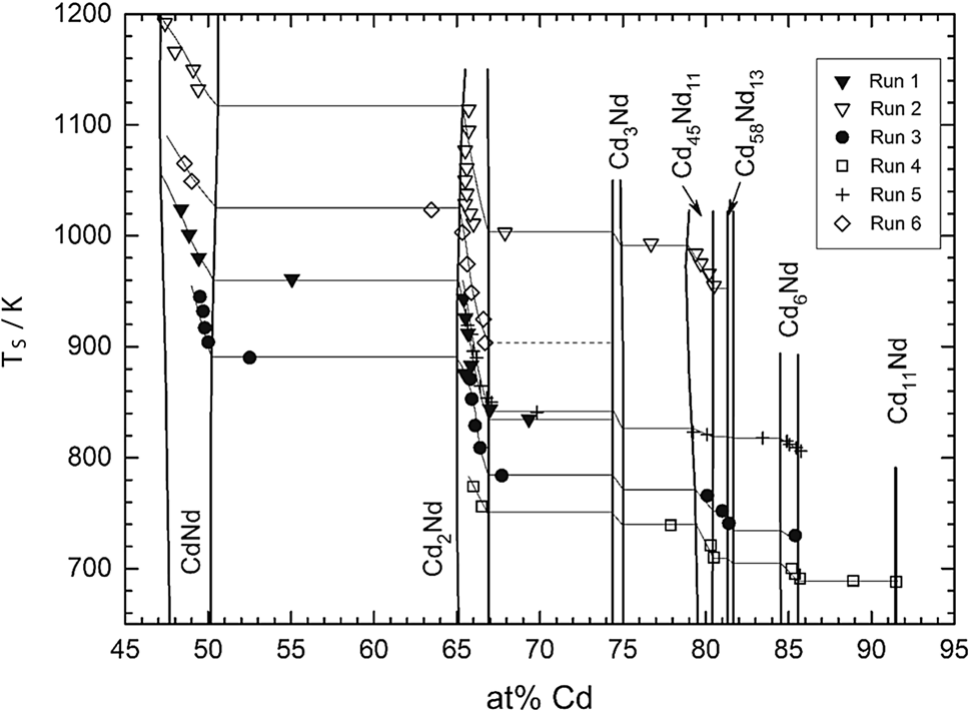
Sample temperature vs. sample composition superimposed on the partial Cd–Nd phase diagram (the *dashed line* in two-phase field Cd_2_Nd + Cd_3_Nd is estimated and not supported directly by data points)

The experimental results, i.e., sample temperature, sample composition, and thermodynamic activity of Cd for each sample, are listed in Table 1. In Fig. 1, sample temperatures are plotted against sample compositions for all experimental runs (the so-called equilibrium curves), superimposed on a partial phase diagram according to Skołyszewska-Kühberger et al. [15]. To check the compositions calculated from the weight change, selected samples were analyzed by energy dispersive X-ray spectroscopy (EDS). In general, the compositions agreed within 0.5 at %, and the temperatures are assumed to be accurate within ±2 K. As can be seen from Fig. 1, the equilibrium samples obtained in the experimental runs cover the concentration range between about 48 and 92 at  % Cd.

**Table 1:** Isopiestic experimental results; standard state: Cd(l)

Sample no.	at % Cd	*T*_S_/K	ln*a*_Cd_ (*T*_S_)	Phases	$\Delta \bar{H}_{\text{Cd}}$/kJ g-atom^−1^	ln*a*_Cd_ (873 K)
Run 1	*T*_R_ = 770 K, 26 days					
1	69.3	835	−1.24	Cd_2_Nd + Cd_3_Nd	−4.3	−1.21
2	67.0	844	−1.39	Cd_2_Nd	−4.3	−1.37
3	65.5	876	−1.92	Cd_2_Nd	−12.0	−1.93
4	65.9	883	−2.03	Cd_2_Nd	−8.8	−2.05
5	65.7	912	−2.47	Cd_2_Nd	−10.3	−2.53
6	65.5	926	−2.67	Cd_2_Nd	−12.0	−2.76
7	65.4	943	−2.91	Cd_2_Nd	−13.1	−3.04
8	55.1	961	−3.15	CdNd + Cd_2_Nd	−20.8	−3.41
9	49.4	980	−3.39	CdNd	−23.5	−3.74
10	48.9	1001	−3.65	CdNd	−27.0	−4.12
11	48.4	1024	−3.92	CdNd	−37.0	−4.67
Run 2	*T*_R_ = 893 K, 33 days					
1	80.5	955	−0.88	Cd_45_Nd_11_	3.3	−0.84
2	80.2	966	−1.03	Cd_45_Nd_11_	2.5	−0.99
3	79.7	975	−1.14	Cd_45_Nd_11_	1.4	−1.12
4	79.4	984	−1.25	Cd_45_Nd_11_	0.8	−1.24
5	76.7	993	−1.36	Cd_3_Nd + Cd_45_Nd_11_	0.6	−1.36
6	67.9	1003	−1.49	Cd_2_Nd + Cd_3_Nd	−4.3	−1.56
7	66.0	1011	−1.58	Cd_2_Nd	−8.2	−1.73
8	65.8	1020	−1.68	Cd_2_Nd	−9.5	−1.87
9	65.5	1029	−1.79	Cd_2_Nd	−12.1	−2.04
10	65.6	1038	−1.89	Cd_2_Nd	−11.1	−2.13
11	65.5	1050	−2.02	Cd_2_Nd	−12.0	−2.30
12	65.6	1061	−2.14	Cd_2_Nd	−11.1	−2.41
13	65.5	1077	−2.31	Cd_2_Nd	−12.1	−2.62
14	65.7	1095	−2.49	Cd_2_Nd	−10.3	−2.78
15	65.7	1114	−2.67	Cd_2_Nd	−10.3	−2.98
16	49.4	1132	−2.84	CdNd	−23.5	−3.59
17	49.1	1150	−3.01	CdNd	−25.3	−3.85
18	48.0	1166	−3.15	CdNd	−44.3	−4.68
19	47.4	1192	−3.37	CdNd	−52.0	−5.29
Run 3	*T*_R_ = 708 K, 32 days					
1	85.4	730	−0.53	Cd_6_Nd	13.7	−0.90
2	81.4	741	−0.78	Cd_58_Nd_13_	5.2	−0.90
3	81.0	752	−1.02	Cd_45_Nd_11_ + Cd_58_Nd_13_	3.3	−1.09
4	80.1	766	−1.32	Cd_45_Nd_11_	2.3	−1.36
5	67.7	784	−1.69	Cd_2_Nd + Cd_3_Nd	−4.3	−1.62
6	66.4	809	−2.17	Cd_2_Nd	−6.2	−2.10
7	66.1	829	−2.53	Cd_2_Nd	−7.7	−2.48
8	65.9	853	−2.95	Cd_2_Nd	−8.8	−2.92
9	65.8	871	−3.24	Cd_2_Nd	−9.5	−3.24
10	52.5	890	−3.54	CdNd + Cd_2_Nd	−20.8	−3.59
11	50.0	904	−3.75	CdNd	−21.2	−3.85
12	49.8	917	−3.94	CdNd	−21.8	−4.09
13	49.7	932	−4.15	CdNd	−22.1	−4.35
14	49.5	945	−4.33	CdNd	−22.9	−4.57
Run 4	*T*_R_ = 687 K, 55 days					
1	91.5	688	−0.03	Cd_11_Nd	14.9	−0.58
2	88.9	689	−0.05	Cd_6_Nd + Cd_11_Nd	14.9	−0.60
3	85.7	691	−0.10	Cd_6_Nd	14.9	−0.65
4	85.4	695	−0.21	Cd_6_Nd	13.7	−0.69
5	85.2	700	−0.33	Cd_6_Nd	12.4	−0.76
6	80.5	710	−0.58	Cd_45_Nd_11_	3.3	−0.69
7	80.3	721	−0.85	Cd_45_Nd_11_	2.7	−0.93
8	77.9	739	−1.26	Cd_3_Nd + Cd_45_Nd_11_	0.6	−1.28
9	66.5	756	−1.64	Cd_2_Nd	−5.8	−1.52
10	66.0	774	−2.02	Cd_2_Nd	−8.2	−1.87
Run 5	*T*_R_ = 781 K, 34 days					
1	85.7	806	−0.49	Cd_6_Nd	14.9	−0.66
2	85.5	809	−0.54	Cd_6_Nd	14.4	−0.70
3	85.0	812	−0.60	Cd_6_Nd	11.1	−0.71
4	84.9	815	−0.65	Cd_6_Nd	10.5	−0.76
5	83.4	818	−0.71	Cd_58_Nd_13_ + Cd_6_Nd	8.4	−0.79
6	80.1	821	−0.76	Cd_45_Nd_11_	2.3	−0.78
7	79.3	823	−0.80	Cd_45_Nd_11_	0.7	−0.81
8	69.8	841	−1.12	Cd_2_Nd + Cd_3_Nd	−4.3	−1.10
9	67.1	850	−1.27	Cd_2_Nd	−4.3	−1.26
10	66.8	854	−1.34	Cd_2_Nd	−4.6	−1.32
11	66.4	865	−1.52	Cd_2_Nd	−6.2	−1.51
12	66.2	890	−1.92	Cd_2_Nd	−7.1	−1.93
13	66.0	896	−2.01	Cd_2_Nd	−8.2	−2.04
14	65.9	911	−2.23	Cd_2_Nd	−8.8	−2.28
15	65.7	919	−2.35	Cd_2_Nd	−10.3	−2.42
16	65.5	928	−2.47	Cd_2_Nd	−12.0	−2.57
Run 6	*T*_R_ = 829 K, 28 days					
1	66.7	904	−1.21	Cd_2_Nd	−5.0	−1.24
2	66.6	925	−1.52	Cd_2_Nd	−5.4	−1.56
3	65.9	949	−1.85	Cd_2_Nd	−8.8	−1.95
4	65.6	975	−2.19	Cd_2_Nd	−11.1	−2.35
5	65.3	1003	−2.54	Cd_2_Nd	−14.5	−2.80
6	63.5	1023	−2.77	CdNd + Cd_2_Nd	−20.8	−3.20
7	49.0	1049	−3.07	CdNd	−26.1	−3.67
8	48.6	1065	−3.23	CdNd	−33.0	−4.05

The majority of the samples were single phase, namely CdNd, Cd_2_Nd, Cd_45_Nd_11_, and Cd_6_Nd. As in several other RE-Cd systems [21–23], no single phase samples of Cd_3_Nd could be obtained in any of the runs suggesting that Cd_3_Nd is only slightly more stable than a two-phase mixture of its neighboring compounds. Thus, the activities of Cd in the adjacent two-phase fields Cd_2_Nd + Cd_3_Nd and Cd_3_Nd + Cd_45_Nd_11_ are only slightly different (cf. Fig. 4). Moreover, it was found that a majority of data points fall into the composition range of the phase Cd_2_Nd indicating that this must be one of the relatively most stable compounds in the Cd–Nd system, in agreement with its congruent formation from the liquid [15].

Some of the samples were obtained in various two-phase fields after equilibration. This was probably caused by slight variations in the sample temperatures during equilibration. These samples were quite useful for an estimate of the partial enthalpies of formation of Cd in these two-phase fields.

### Evaluation of the thermodynamic activity of Cd

**Fig. 2:**
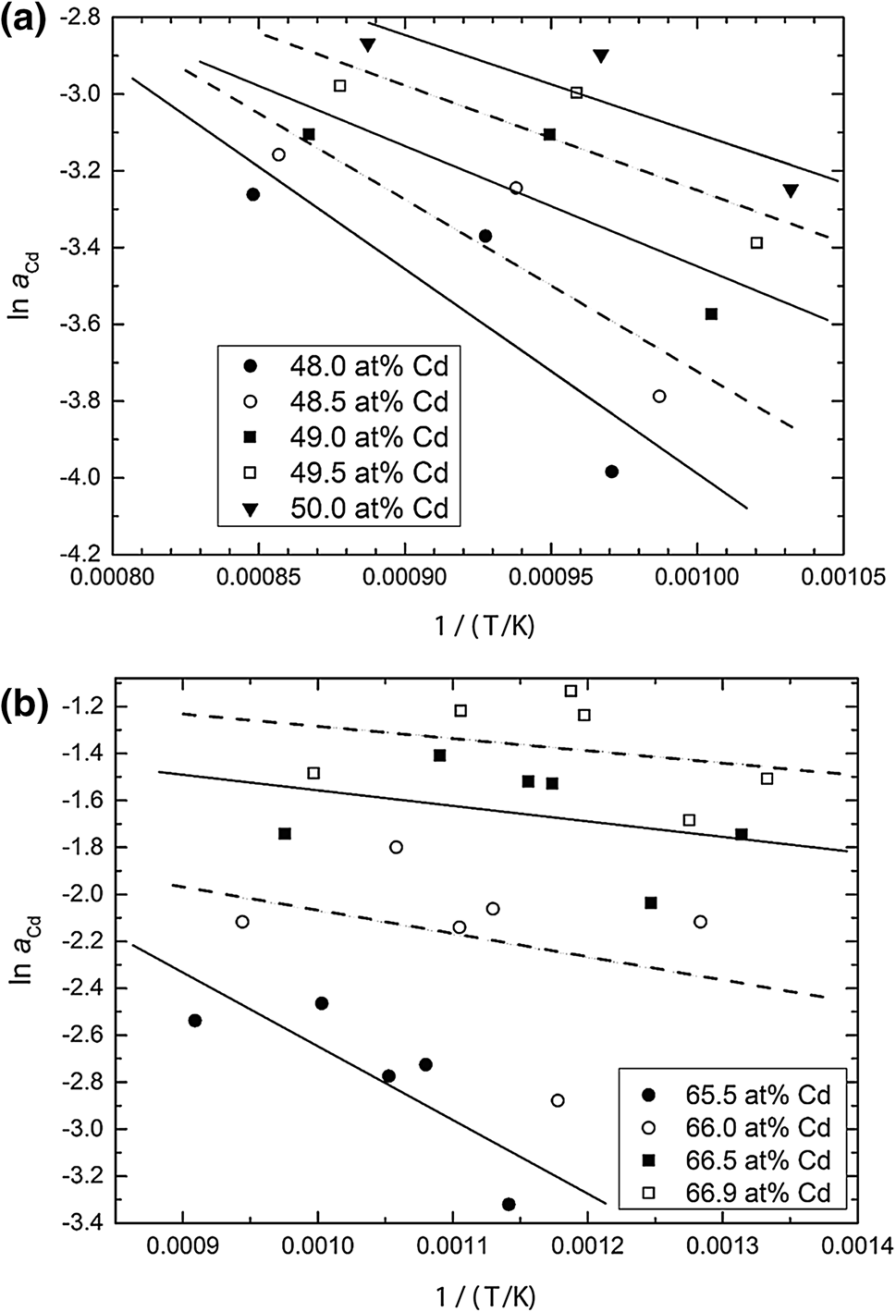
Natural logarithm of the Cd activity vs. reciprocal temperature in the phases CdNd (**a**) and Cd_2_Nd (**b**)

Equation (2) can be used to calculate the thermodynamic activity of Cd for each individual data point in Fig. 1; however, it is obtained exactly for the temperature of the sample. To provide the composition dependence of the activity at one common temperature (*T*_2_), one needs the partial enthalpies of formation of Cd to convert the activity values to this temperature *T*_2_ according to:

4 $$\ln a_{\text{Cd}} (T_{2} )\, - \,\ln a_{\text{Cd}} (T_{1} ) = \frac{{\Delta \bar{H}_{\text{Cd}} }}{R} \times \left( {\frac{1}{{T_{2} }} - \frac{1}{{T_{1} }}} \right)$$

which is an integrated form of the Gibbs–Helmholtz equation. To obtain these $\Delta \bar{H}_{\text{Cd}}$ values, one can use the same equation in its differential form

5 $$\frac{{\partial \,\ln a_{\text{Cd}} }}{\partial (1/T)} = \frac{{\Delta \bar{H}_{\text{Cd}} }}{R}$$

and the partial enthalpy values can be derived from a plot of ln *a*_Cd_ vs. 1/*T* at a given composition. For this purpose, the so-called equilibrium curves (sample temperature vs. sample composition) in Fig. 1 are used: hypothetical sample temperatures are derived for defined compositions within the homogeneity ranges of the various phases from which the Cd activities are calculated according to Eq. (2), and they are plotted versus the reciprocal temperature. This is shown, as an example, for the phases CdNd and Cd_2_Nd in Fig. 2.

**Fig. 3:**
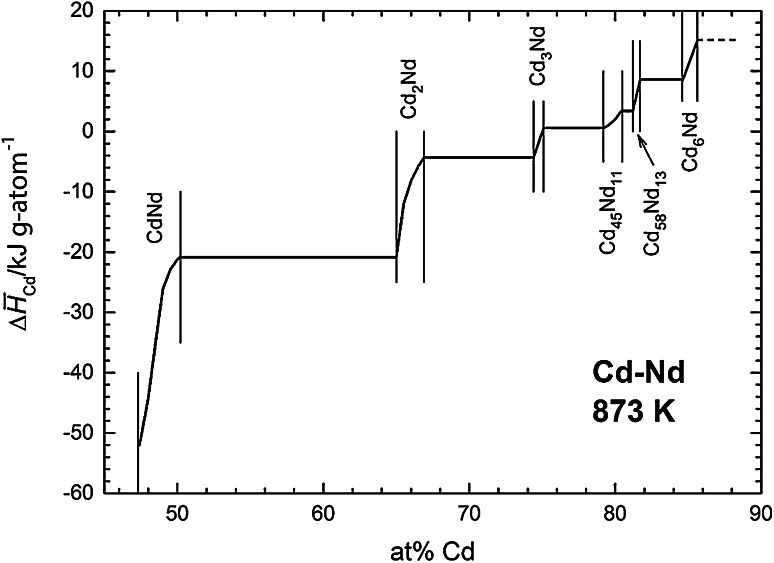
Partial molar enthalpy of Cd in the Cd–Nd system; standard state: Cd(l)

A similar procedure was applied to estimate partial molar enthalpies of formation of Cd in the two-phase fields, assuming that the phase boundaries do not change with temperature (which, of course is not fully correct). For all phase fields where no or not enough data points were available, i.e., for the two single-phase fields of Cd_3_Nd and Cd_58_Nd_13_, a linear variation of $\Delta \bar{H}_{\text{Cd}}$ with composition was assumed. Figure 3 shows the partial enthalpy of formation of Cd as a function of composition between 45 and 90 at % Cd and numerical values from this curve are included in Table 1.

Two things should be pointed out. As can be seen in Fig. 2, the scatter of the data points is considerable which means that the derived $\Delta \bar{H}_{\text{Cd}}$ values will exhibit an appreciable error limit, probably more than ±5 kJ g-atom^−1^. Furthermore, the numerical values of the partial enthalpies of Cd become positive for Cd contents of more than 75 at % Cd. This is somewhat surprising though not impossible since it depends on the shape of the curve of the integral enthalpy of formation.

**Fig. 4:**
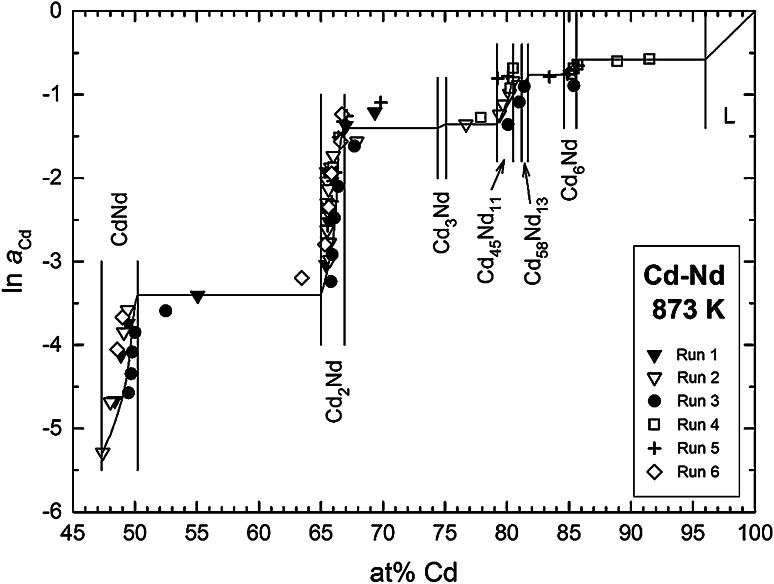
Natural logarithm of the Cd activity in the Cd–Nd system at 873 K; standard state: Cd(l)

Using Eq. (4) and $\Delta \bar{H}_{\text{Cd}}$ values from the curve in Fig. 3, the Cd activities were converted to a temperature of 873 K which is approximately the average temperature of all samples. Using such a mean temperature minimizes any errors that would be introduced by errors in the partial enthalpy values. Figure 4 shows finally a plot of ln *a*_Cd_ as a function of composition for the Cd–Nd system over the entire composition range between 47 and 100 at % Cd. The phase boundary of the liquid phase at 873 K was taken from the phase diagram study by Skołyszewska-Kühberger et al. [15] in agreement with earlier results by Johnson et al. [24].

### Integral Gibbs energy

Rather limited information has been available up to now on the thermodynamics of the Cd–Nd system. To perform a Gibbs–Duhem integration one needs an integration constant, i.e., a value for the integral Gibbs energy or the partial Gibbs energy of Nd at a given composition. Vandarkuzhali et al. [18] determined the integral Gibbs energy of formation of Cd_11_Nd in the temperature range 698–773 K. Unfortunately, 773 K is rather at the lower limit of the present experiments, and only one single data point was obtained for the Cd_11_Nd-phase (see Fig. 1). On the other hand, Cd_11_Nd decomposes in a peritectic reaction at 793 K [15] and does not exist anymore at 873 K, the average temperature of all data points (see above).

**Fig. 5:**
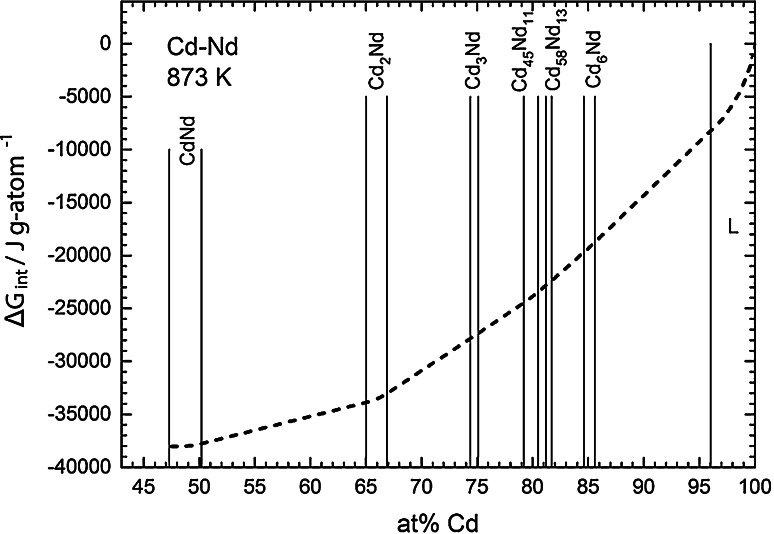
Integral Gibbs energy of formation vs. composition at 873 K in the Cd–Nd system; standard states: Cd(l) and Nd(s)

Therefore, it was decided to use a value of the integral Gibbs energy of formation for Cd_6_Nd that had been derived by Kurata and Sakamura [7] in their optimization of the Cd-rich part of the Cd–Nd system. They reported an equation

6 $$\frac{{\Delta_{\text{f}} G({\text{Cd}}_{ 6} {\text{Nd}})}}{{{\text{J mol}}^{ - 1} }} = - 205,200 + 84.71 \times \frac{T}{\text{K}}$$

which results in a value of −131,248 J mol^−1^ or −18,750 J g-atom^−1^ for a temperature of 873 K. From this, a value of ln *a*_Nd_ = −14.49 was derived for the Cd-rich phase boundary of Cd_6_Nd at *x*_Cd_ = 0.856 at 873 K. Using this value as an integration constant, a Gibbs–Duhem integration was performed based on Darken’s α-function [25] to derive the integral Gibbs energy at 873 K for the composition range between 47 and 100 at % Cd (Fig. 5). This curve should rather be considered as an estimate since any uncertainty of the integration constant will, of course, add to the uncertainty of the present data. Therefore, it is shown as a dashed line in Fig. 5. According to these calculations, the most stable compound in the system is CdNd with Δ_f_*G* ≈ −38 kJ g-atom^−1^. This is well comparable with the corresponding Gibbs energies for other CdRE compounds, i.e., CdCe (−37 kJ g-atom^−1^) [21], CdPr (−35 kJ g-atom^−1^) [23], and CdGd (−34 kJ g-atom^−1^) [22]. Finally, Table 2 lists smoothed values of the Cd and Nd activities as well as of the integral Gibbs energies over the entire investigated composition range.

**Table 2:** Smoothed values of the Cd and Nd activities and of the integral Gibbs energy at 873 K in the Cd–Nd system; standard states are Cd(l) and Nd(s)

At % Cd	Phase	ln *a*_Cd_	ln *a*_Nd_	Δ*G*_int_/kJ g–atom^−1^
100.0	L	0.00	−∞	0.00
98.00	L	−0.29	−25.36	−5.7
96.00	L/L + Cd_6_Nd	−0.58	−14.49	−8.3
85.60	L + Cd_6_Nd/Cd_6_Nd	−0.58	−14.49	−18.8
85.00	Cd_6_Nd	−0.70	−13.79	−19.3
84.60	Cd_6_Nd/Cd_6_Nd + Cd_58_Nd_13_	−0.76	−13.46	−19.7
81.80	Cd_6_Nd + Cd_58_Nd_13_/Cd_58_Nd_13_	−0.76	−13.46	−22.3
81.50	Cd_58_Nd_13_/Cd_58_Nd_13_ + Cd_45_Nd_11_	−0.84	−13.10	−22.6
80.40	Cd_58_Nd_13_ + Cd_45_Nd_11_/Cd_45_Nd_11_	−0.84	−13.10	−23.5
80.00	Cd_45_Nd_11_	−1.09	−12.09	−23.9
79.20	Cd_45_Nd_11_/Cd_45_Nd_11_ + Cd_3_Nd	−1.36	−11.04	−24.5
75.00	Cd_45_Nd_11_ + Cd_3_Nd/Cd_3_Nd	−1.36	−11.04	−27.4
74.40	Cd_3_Nd/Cd_3_Nd + Cd_2_Nd	−1.40	−10.92	−27.8
66.90	Cd_3_Nd + Cd_2_Nd/Cd_2_Nd	−1.40	−10.92	−33.0
66.50	Cd_2_Nd	−1.64	−10.44	−33.3
66.00	Cd_2_Nd	−2.73	−8.30	−33.6
65.50	Cd_2_Nd	−3.13	−7.53	−33.7
65.00	Cd_2_Nd/Cd_2_Nd + CdNd	−3.40	−7.02	−33.9
50.20	Cd_2_Nd + CdNd/CdNd	−3.40	−7.02	−37.8
50.00	CdNd	−3.52	−6.90	−37.8
49.50	CdNd	−4.30	−6.22	−38.0
49.00	CdNd	−4.62	−5.82	−38.0
48.50	CdNd	−4.87	−5.58	−38.0
48.00	CdNd	−5.08	−5.39	−38.0
47.40	CdNd/CdNd + β-Nd	−5.29	−5.20	−38.0

## Conclusion

Thermodynamic activities of Cd were determined for the Cd–Nd system in the temperature range between about 690 and 1200 K and the composition range between 48 and 92 at % Cd based on an isopiestic vapor pressure method. Partial enthalpies of formation of Cd were derived from the temperature dependence of the activity. These data were used to convert the activity values to a common temperature of 873 K. Using a literature value of Δ_f_*G* for Cd_6_Nd as integration constant, it was possible to calculate Nd activities and integral Gibbs energies of formation at 873 K for the same composition range. A minimum of Δ_f_*G* ≈ −38 kJ g-atom^−1^ was obtained in the phase CdNd.

## Experimental

The principle and experimental details of the isopiestic method applied in this work were described previously by Ipser et al. [5, 6]. A schematic diagram of the particular setup used in the present investigation has been shown, for example, by Skołyszewska-Kühberger et al. [21] and Reichmann et al. [22]. The apparatus is essentially made of quartz glass. It consists of an outer tube of 38 mm OD with one end closed and the other end fitted with a ground joint which can be connected to a vacuum pump. A quartz glass crucible with 32 mm OD is placed at the bottom, serving as a reservoir for Cd. On top of the reservoir, a quartz glass spacer of suitable height and a quartz supporting tube (15 mm OD) are located where the tantalum crucibles containing pure Nd as samples are inserted. An inner tube of 7 mm OD with its upper end widened to 32 mm OD is used as a thermocouple well. The apparatus can be sealed under vacuum in its upper part.

Before use the entire apparatus was cleaned with an acid mixture (HF/HNO_3_/H_2_O), rinsed with distilled water, and dried. Afterward the fully assembled setup, including the empty tantalum crucibles (approximately 20), was degassed under vacuum (10^−3^ mbar) at 900 °C for 5 h. All preparations for the experiments were then carried out under Ar atmosphere in a glove box. The reservoir was filled with 25–35 g of Cd (99.9999 % Alfa AESAR, Karlsruhe, Germany), depending on the experimental reservoir temperature. Between 150 and 200 mg of pure Nd (99.9 % Alfa AESAR, Karlsruhe, Germany, and smart-elements, Vienna, Austria) were weighed into each Ta crucible with an accuracy of ±0.1 mg. The assembled apparatus was brought outside the glove box securely closed using a vacuum valve suitable to directly connect to a vacuum pump. The apparatus was then evacuated and sealed under a dynamic vacuum of better than 10^−3^ mbar.

The isopiestic equilibration experiments were carried out in different temperature gradients, applied by two-zone furnaces, for periods of about 4–8 weeks depending on the respective reservoir temperature. The temperatures of the samples (*T*_S_) and the reservoir (*T*_R_) were measured periodically by raising a Pt/Pt 10 % Rh thermocouple inside the thermocouple well. After equilibration, the isopiestic apparatus was quenched in cold water and cut open in air by a diamond saw. The individual samples (which had become Cd–Nd alloys during the equilibration) together with the crucibles were weighed in air. Immediately afterward they were brought into the glove box and weighed once again. No significant mass difference could be detected in any of the samples, and the sample compositions were derived from the mass difference before and after equilibration that was attributed to the uptake of Cd.

Representative samples were characterized by XRD with Cu Kα radiation on a Bruker D8 Advance Diffractometer with Bragg–Brentano pseudo-focusing geometry. Rietveld refinement was done by means of the TOPAS 3 software (provided by Bruker), making a full pattern refinement with empirical peak profile modeling. To check the calculated compositions, selected samples were also analyzed by energy dispersive X-ray spectroscopy (EDS) in a Zeiss Supra 55 VP scanning electron microscope (SEM).

## Footnotes

1 *CALPHAD* CALculation of PHAse Diagrams.
